# Case of Ovariotomy

**Published:** 1873-03-15

**Authors:** J. T. Everett

**Affiliations:** Sterling, Ill.


### Original Communications.

### CASE OF OVARIOTOMY.

BY J. T. EVERETT, A.M., M.D., STERLING, ILL.

Was called, Nov. 7th, in consultation with Drs. Hunt and Newton, to see Mrs. F., aged forty-one years ; American by birth ; bilious temperament and full habit.

Found patient complaining of a lump in left ovarian region, which had been observable for some time, slowly increasing in size. Although still comparatively small, it gave rise to a good deal of mental trouble, from the fear that it might be a malignant growth. Upon careful examination, we became convinced that it was an encysted ovarian tumor.

After completing the diagnosis, the nature of the trouble was explained to the patient and friends, and an early operation advised. Accordingly, the 17th of the same month was fixed for the removal, and the patient placed upon the use of tinct. ferri chlorid., 10 gtts., three times a day, to render the blood more plastic, and to prevent any probable chance of cepticemia, or erysipelas, following the operation. Upon the 16th a free purge was given, producing a thorough evacuation ; and an anodyne was then given, to quiet any irritation which the purge might have set up.

After having taken these precautionary measures, I proceeded to operate in the following manner :

The patient was fully anæsthetised with sulph. ether. Then an incision, three inches in length, was made along the linea alba, extending from the pubis upward. After having divided the integument and superficial fascia, the aponeurosis of the rectus muscles was carefully divided, until the peritoneal sac was reached. This, having been carefully divided, and the sac carefully separated from the surrounding tissues, the large trochar, with hose attached, was thrust into the cyst, and the fluid (which consisted of eight and three-fourths pounds of dark colored serum, containing flakes of fibrine and only a small quantity of albumen), was drawn off. I next seized the sac, and, withdrawing it through the opening as far as possible, proceeded to ligate it with a double carbolized animal ligature, thrusting the needle through the center of the pedicle and tying both ways. Then trimmed the stump as close as was deemed safe, and washed out the wound with carbolated artificial serum, and replaced the stump. The wound was closed with the quilled suture of silver wire, and over all placed a film of carbolated colodion. Collecting and weighing the sac and contents, we found it to weigh ten and one-half pounds. I now left the patient under the care of the family physician, Dr. Hunt, from whose notes I draw the subsequent history of the case. For the first four or five days, the patient was kept fully under the influence of an opiate, and 20 gtts. of the tinct. ferri chlorid., given three times a day. On the 23d, the bowels were moved by an enema. On the 24th, there was a slight appearance of puffiness at the lower portion of the wound, and upon the removal of the film of colodion, about four ounces of thin serum escaped. An injection of carbolated water was thrown into the cavity, thoroughly washing it out, after which it was closed again with carbolated oil and lint dressing. From this time on the wound was washed out daily, the cavity steadily lessening in size, until, at the end of the third week, there remained but a small, fistulous orifice the size of a crow-quill ; which had entirely closed up by the end of the fourth week from the time of the removal.

No signs of peritonitis appeared at any time, and the patient made a rapid and permanent recovery. The carbolized ligatures never came out, and must have been either encysted, or, what is more probable, absorbed. The ligatures were made of an E violin string, soaked in ninety-five per cent. solution of carbolic acid.

The result was in every way satisfactory, and the patient is now doing her own work, and claims to be as well as ever.

I report this case, more to show the rapid recovery from, and comparative simplicity, of the operation, when performed in the early stage of the disease, than from any desire to show surgical skill or originality in procedure.

I believe that ovariotomy, if performed early, is as devoid of danger of serious results as is an amputation of the hand.

